# Correction: An early endosome–derived retrograde trafficking pathway promotes secretory granule maturation

**DOI:** 10.1083/jcb.20180801712202021c

**Published:** 2022-02-16

**Authors:** Cheng-I J. Ma, Yitong Yang, Taeah Kim, Chang Hua Chen, Gordon Polevoy, Miluska Vissa, Jason Burgess, Julie A. Brill

Vol. 219, No. 3 | 10.1083/jcb.201808017 | February 11, 2020

The initial published version of Table S1 included a Rab1 RNAi line, P{TRiP.JF02609}attP2. That line was not tested in the RNAi screen and has been removed from the supplemental file. The authors apologize for any confusion. This error appears only in versions of Table S1 downloaded on or before January 26, 2022.

